# Does social cohesion mediate neighbourhood effects on mental and physical health? Longitudinal analysis using German Socio-Economic Panel data

**DOI:** 10.1186/s12889-020-09149-8

**Published:** 2020-07-01

**Authors:** Sara Kress, Oliver Razum, Kim Alexandra Zolitschka, Jürgen Breckenkamp, Odile Sauzet

**Affiliations:** 1 Department of Epidemiology and International Public Health, School of Public Health, Bielefeld University, Bielefeld, Germany; 2 Centre for Statistics, Bielefeld University, Bielefeld, Germany

**Keywords:** Neighbourhood effects, Social mechanism, Social cohesion, Mediation

## Abstract

**Background:**

Neighbourhood has risen as a relevant determinant of health. While there is substantial evidence that environmental factors affect health, far less evidence of the role of social mechanisms in the causal chain between neighbourhood characteristics and health is available.

**Method:**

To evaluate the role of social cohesion as a mediator between four different neighbourhood characteristics and health using data from German Socio-Economic-Panel (SOEP), a longitudinal mediation analysis was performed. Multilevel linear regression models adjusted for socio-economic variables involved three time points and two measures of physical and mental health (physical and mental component scores (PCS and MCS) of the SF12 Questionnaire. Participants were followed-up for 4 and 10 year starting in 2004.

**Results:**

A total of 15,518 measures of MCS and PCS on 10,013 participants living in 4985 households were included. After adjusting for values of MCS and PCS at baseline and demographic/socio-economic variables, social cohesion was a significant positive predictor of both MCS and PCS (β-coefficient MCS: 1.57 (0.27); PCS: 1.50 (0.24)). Interaction between social cohesion and follow-up were significant for PCS. The effect of environmental and built characteristics on health was consistently mediated by social cohesion with proportion varying between 10 and 23%.

**Discussion:**

We show that social cohesion is part of the causal chain between environmental and built characteristics of a neighbourhood and health, with increasing mediation effect over time for physical health. Social mechanisms should be considered when studying the effect of neighbourhood characteristics on health inequalities making social cohesion as a legitimate target of public health interventions at neighbourhood level.

## Background

A neighbourhood can be considered as a small-area unit in which inhabitants are exposed to similar contextual environmental characteristics, and in which they interact socially [1]. In the past decades, neighbourhood has risen as a relevant determinant of health studied in epidemiology with a large body of publications available on contextual effects on health inequalities. However, the effect of social interactions within the neighbourhood on health is still largely at the stage of hypothesis [2] and evidence of causal relationship is weak [3, 4] – making neighbourhood with regard to its effects on health literally a “black box”.

Van Ham and Manley [5] have pointed out the necessity of explaining what is in the “black box” of neighbourhood effects moving away from association studies to studies which elucidate the causal mechanisms connecting neighbourhood and health. In particular, the role of social factors such as social norms, social contagion, or social cohesion, within a neighbourhood should be better understood. Moreover, quantitative evidence of dose-response effects should be obtained [3]. Improving the understanding of the complex aetiology of neighbourhood effects on health could have implications in terms of public health interventions toward the reduction of health inequalities [6].

The work of Sampson in the 1990s has emphasised the role of neighbourhood social cohesion in the context of deprivation and has shown that the lack of social cohesion leads to increased mental distress [7–10]. Several studies indicate a mediating role between socio-economic status and mental health [11] .

Social cohesion is a sociological concept, which encompasses social bonds among inhabitants of a neighbourhood. Its operationalization in studies on neighbourhood effects on health inequalities has been varied and includes questions on trust of neighbours or their perceived availability for various tasks (Sampson, 1997), knowledge of neighbours, or feeling of safety [12]. In the literature, social cohesion is usually operationalized, as perceived individual-level factors, which are subjective in nature and not as an objective small-area aggregated characteristic which would provide a measure common to those sharing a neighbourhood.

Recent studies have shown a mediating role of social cohesion on the effect of particular neighbourhood characteristics such as streetscape greenery [12], socio-economic status and ethnic composition [13] on measures of general health. But all of these studies used cross-sectional data, which limits the possibility to assess the role of social cohesion in the causal chain. Moreover, studies focused just on one aspect of neighbourhood characteristics.

We aim to establish the role of social cohesion in the causal chain between different types of urban neighbourhood characteristics and a general measure of physical and mental health over time. In particular, we wanted to answer the following questions:

- Does social cohesion mediate the effects of environmental factors (pollution, noise), built environment, accessibility of public transport, and accessibility to other infrastructures (schools, shops, etc.) on physical and mental health 4 and 10 years later?
- Does the mediation effect change over time?
- Does the mediation effect depend of the type of neighbourhood characteristics?

## Methods

We performed a longitudinal mediation analysis using three time points and two measures of physical and mental health to evaluate the role of our measure of social cohesion as a mediator between perceived neighbourhood characteristics and health and to quantify its share of associations between neighbourhood characteristic and health [14].

### Sample

The analysis is based on data from the German Socio-Economic Panel (SOEP), a household panel run on behalf of the German Institute for Economic Research. Some 15,000 households including over 25,000 individuals are surveyed every year on a range of topics as employment, education, values and health (health: every 2 years). Regional representativeness is attained for highly populated regions [15, 16].

We investigated the role of social cohesion as a mediator of neighbourhood characteristics measured in 2004 on the health of the participants living in urban areas over two different time periods (2004–2008; 2004–2014).

### Outcome

A general measure of health was used in the form of the mental (MCS) and physical (PCS) component of the SOEP version of the SF12 questionnaire [17]. The SF12 is the short version of the SF36 with 12 questions on health and well-being, covering the two dimensions on physical and mental health. The SOEP version differs from the original SF12 only in one question, and the dimensions were validated for the German population using SOEP data for 2004 (see [17] for more details on validation and how the scores are built). The standardised scales have a mean of 50 and a standard deviation of 10. On both scales, higher values represent better health.

### Operationalisation of social cohesion

We developed a composite measure of perceived social cohesion based on the literature by taking the means of following relevant variables measured on a Likert scale available in the SOEP survey:

importance of being socially and politically active as an aspect of participation in the neighbourhood’s life [18],

being worried about crime in the neighbourhood as an aspect of trust [3, 19],

being worried about the hostility to foreigners as an aspect of tolerance or respect at neighbourhood level [20] .

A higher value of the measure corresponds to higher social cohesion.

### Operationalisation of neighbourhood characteristics

Four types of neighbourhood characteristics were operationalised as composite measures based on literature according to the perceived contextual characteristics [21, 22] reported by the survey respondents. Each of these aspects was summarised into a quasi-metric score as above.

A measure of environmental characteristics was obtained as a mean between bothersome noise pollution, bothersome air pollution, and shortage of green areas [3, 21, 23].

A measure of the built characteristics was obtained by including variables relative to housing characteristics [21, 22, 24]. These include questions about the type of area (e.g. Industrial, residential), type of houses (e.g. High rise, single family houses) and the state of repairs. A measure of the geographical characteristics concerned the walking distance to public transport, and distance to work [3] while a measure of the institutional characteristics included walking distance to shops, bank, doctor, nursery, primary school, secondary school, youth meeting place, elderly facility, and to sporting facilities [3, 21].

### Mediation analyses

The aim of a mediation analysis is to assess the role of a variable (here social cohesion) as a mechanism in the association between two variables (here neighbourhood characteristic and health) between which a causal pathway is assumed. The total effect of the neighbourhood characteristics on health (measured in 2008 and 2014) was obtained by regressing MCS and PCS respectively on the four measures of neighbourhood characteristics (measured in 2004) (Reg. 1). This total effect was decomposed into a direct effect of neighbourhood characteristic on MCS and PCS by regressing MCS and PCS respectively on the four measures of neighbourhood characteristics and social cohesion (Reg. 2); and into an indirect effect (α훽) of neighbourhood characteristic via our measure of social cohesion on health outcomes [14]. The α-coefficients are the regression coefficients of neighbourhood characteristics obtained by regressing social cohesion on neighbourhood characteristics (Reg. 3). The β-coefficient is the regression coefficients of social cohesion in Reg. 2. The statistical significance of the indirect effect was tested using the Sobel test [25]. The share of mediation was obtained as the relative part of the indirect effect to the total effect.

The two follow-up points in time were included in one multilevel model to adjust for repeated measures. The statistical significance of time effects on mediation was tested by estimating interaction between follow-up time and either neighbourhood characteristics or social cohesion when appropriate. The analysis was performed using R [26].

All regression models performed in the mediation analysis where controlled for the following individual level confounders: sex, age, born in Germany, household equivalent income, education, employment status, and marital status measured in 2004. Moreover we also adjusted respectively for the MCS or PCS measured in 2004. We used multilevel models to adjust for the non-independence of respondents living in the same household and for the year of measurement of health outcome. A sensitivity analysis was performed by including the following variables into the models: federal state, residential move.

## Results

### Participants

Out of 22,012 persons who participated in survey year 2004, 16,018 persons (72.8%) participated in survey year 2008 as well. After restricting the sample to the participant living in an urban area for which the SF-12 is available, 10,665 persons for the period between 2004 and 2008 were included in the analysis and 6083 persons for the years 2004 and 2014. During the period 2004–2008, 5.4% of respondents moved to another area and 8.7% in the period 2004–2014. We present descriptive statistics separated by subsamples of participants with SF-12 data available for 2008 and 2014 separately in Table 1. The proportion of participants living in poverty or having a low level of education seems higher among the subsample with health data available in 2008 than among the subsample of those with health data in 2014. This might be because the younger participants were still in education in 2008 but also mostly that these population groups are more likely to stop participating in the panel study.

**Table 1:** Descriptive statistics of the subsamples with SF-12 available for 2004, 2008 and one for 2014. Values are provides as mean (standard deviation) or frequency (percentage from the total available for this year)

SF12 available for:	2008 *N* = 10,665	2014 *N* = 6083
Socio-demographic variables measured in 2004		
Male	5011 (47%)	2824 (46%)
Age	51.5 (16.6)	57.3 (15.1)
Non- German nationality	905 (8.5%)	395 (6.5%)
Household income under the poverty line	2907 (27%)	2907 (28%)
Education lower than high school	1613 (16%)	726 (12%)
Married	3811 (36%)	2067 (34%)
Holding a job	10,031 (94%)	5727 (94%)
Health outcome (SF-12) measures in 2004, 2008 and 2014 respectively		
Mental component	50.7 (9.9)	50.7 (10.1)
Baseline values (2004)	49.2 (13.0)	49.4 (12.5)
Physical component	49.1 (10.1)	47.5 (10.2)
Baseline values (2004)	49.0 (13.1)	49.6 (12.3)
Neighbourhood characteristics measured in 2004		
Environmental	3.31 (0.66)	3.32 (0.65)
Built	2.54 (0.47)	2.54 (0.47)
Geographical	3.54 (0.60)	2.19 (1.26)
Institutional	3.01 (0.70)	3.03 (0.68)
Social cohesion	2.03 (0.36)	2.04 (0.35)

From the eligible sample in 2004, 297 persons failed to answer the health questions in 2008 and 422 persons in 2014. The average mental health score remained stable over time while the average physical health score declined.

Our measure of perceived social cohesion provided a score with a symmetric distribution with a mean of 2.03 (standard deviation: 0.36). This measure is constant across the samples with measures available in 2008 and with measures available in 2014 Table 1). Measures of neighbourhood characteristics remained also constant, with the exception of the geographical characteristics, which also showed more variability than the other characteristics.

### Mediation analyses

The mediation analysis included 15,518 measures of MCS and PCS (for 2008 and 2014) on 10,013 participants living in 4985 households. Social cohesion was a significant positive predictor of both MCS and PCS (β-coefficient MCS: 1.57 (Standard error: 0.25); PCS: 1.50 (0.22)). Interactions with follow-up time and social cohesion were not significant for MCS but significant for PCS. None of the associations between neighbourhood characteristics and health had significant interactions with follow-up time. In consequence, we present results of the mediation analyses over the two follow-up periods without interaction terms for MCS and with interaction terms for PCS, providing separate mediation analyses for the two follow-up times. The results of all regression models are given in the supplementary material. Results of the mediation analyses are given in Table 2.

**Table 2:** Results of the mediation analysis

Neighbourhood characteristic	Direct effect	Indirect effect	Total effect	t-statistic Sobel test	*p*-value Sobel test	Proportion mediation
**Mental health**	2004–2008-2014					
β-coefficient (mediator: social cohesion):				1.93^a^		
Environmental	1.21^a^	0.16^b^	1.37^a^	6.50	< 0.001	0.12
Built	0.87^a^	0.13^b^	1.00^a^	5.31	< 0.001	0.13
Geographical	0.25^a^	0.00	0.25^a^	0.31	0.38	–
Institutional	0.60^a^	−0.03^b^	0.57^a^	−2.18	0.02	0.04
**Physical health**	2004–2008					
β-coefficient (mediator: social cohesion):				1.75^a^		
Environmental	0.71^a^	0.15^b^	0.87^a^	4.03	< 0.001	0.17
Built	1.07^a^	0.12^b^	1.20^a^	3.46	< 0.001	0.10
Geographical	0.10	0.00	0.10	0.23	0.38	–
Institutional	0.61^a^	−0.02^b^	0.59^a^	−1.57	0.06	–
**Physical health**	2004–2014					
β-coefficient (mediator: social cohesion):				2.38^a^		
Environmental	0.71^a^	0.20^b^	0.91^a^	5.48	< 0.001	0.23
Built	1.07^a^	0.16^b^	1.21^a^	4.71	< 0.001	0.13
Geographical	0.10	0.00	0.10	0.31	0.38	–
Institutional	0.61^a^	−0.03^b^	0.59^a^	−2.13	0.02	0.05

N: 15518 measures of health on 10,013 participants

^a^significant at 5% level

^b^α-coefficient significant at 5% level. Measure for physical and mental health provided by the SF-12 questionnaire

Social cohesion mediated positively the effect of both environmental and built environment characteristics on MCS and PCS with all direct effects, total effects, and α-coefficients being positive and significant at the 5% level. The Sobel test for the mediation effect showed high significance and the proportion of mediations ranged from 11 to 25% with the effect of environmental characteristics on PCS being the most mediated (18% in 2008 and 25% in 2014). There were no significant effects of geographical characteristics on MCS and PCS. The effects of institutional characteristics on MCS and PCS were negatively mediated by social cohesion and the proportion of mediation was small in both cases (4–5%). None of the mediations were full mediations; in other words, the effects of neighbourhood characteristics remained significant after adding social cohesion in the model.

For PCS there was a significant (through a significant change of the effect of social cohesion on PCS over time) increase in the proportion of mediation from 2008 to 2014 for environmental and built characteristics; the part of the effects being mediated increased from 18 to 25% (environment) and from 11 to 16% (built).

The sensitivity analysis showed that adjusting for additional individual-level factors did not affect the results of the mediation analyses, and that the reported neighbourhood characteristic and social cohesion changed only marginally over time. The estimates obtained from the regression analysis are provided in supplementary material.

## Discussion

This work provides for the first time longitudinal evidence of the role of social cohesion in the assumed causal pathway between a range of neighbourhood characteristics and physical and mental health. We could base our analysis on longitudinal data with three time points, thus responding to calls made in the literature for providing quantitative evidence for a possible causal effect [3, 5]. Our operationalisation of perceived social cohesion partially mediated the associations between self-reported environmental and build neighbourhood characteristics on the one hand, and physical as well as mental health on the other. The effects of self-reported geographical characteristics on mental health were not mediated by social cohesion, and we observed no significant effect on physical health. Institutional neighbourhood characteristics were only weakly negatively mediated. While we could not see a change in mediation over time for mental health, the mediating role of social cohesion increased between 2008 and 2014 for physical health.

Only the effect of neighbourhood characteristics, which correspond to the immediate environment of respondents including noise, air pollution, or quality of the built environment – as opposed to distances to amenities – seems to be mediated by social cohesion. Having difficulties to reach work or public transport does not seem to show a strong effect on health in this collective. While distance to amenities does have an effect on health, this effect is either not – or only to a small extent – mediated by social cohesion.

An important aspect of gathering evidence about the causal nature of observed associations is whether a dose-response is present [3]. While the mediation effect of social cohesion was stable over time for mental health, we could show that for physical health, the relative part of social cohesion in the effect of environmental characteristic did increase over time, which can be seen as an aspect of dose-response effect. Among the respondents of the study, however, the perception of these characteristics did not change over time. This indicates that a cumulative effect of the neighbourhood characteristics on health may be increasingly due to a cumulative effect of social cohesion indicating that we may have a dose-response effect of social cohesion on health.

We conclude that a substantial part of the observed effects of neighbourhood characteristics are due to social factors. This has consequences for research into the neighbourhood effect in small-area health inequalities as it shows the limitations of considering physical characteristics separately for the social environment. While measuring noise or the quality of the built environment is relatively easy, the operationalisation of social cohesion or other social factors remains challenging. So far, unified instruments are lacking which take into account perceived as well as more objective measures, and which would allow comparisons across studies.

### Strength and limitations

To our knowledge, this is the first longitudinal analysis of the mediation role of social cohesion in neighbourhood effect studies on health inequalities using a validated measure of general health over two repeated measures considering three times points. We also considered a range of neighbourhood characteristics so that we were able to draw conclusion about which type of characteristics have an effect more likely to be mediated by social cohesion. This work is based on over 10,000 respondents in a representative panel study which covers all urban areas of the most populated western European country. A limitation of the conclusions that we can draw is that we used pseudo-metric scales which limits the reliability of the regression models used. Moreover, we only included perceived measured of neighbourhood characteristics and of social cohesion, so that a part of the observed association may be due to the observer. More precisely the health status might influence how one perceive the neighbourhood characteristics as well as social cohesion within the neighbourhood. However, perceived characteristics as opposed to measured characteristics are important determinants of health in their own right and they have the advantage of not having to use predefined administrative neighbourhoods [5]. While we adjusted all our models for socio-economic and demographic variables, the association observed may still be confounded by unmeasured factors.

## Conclusion

We show that social cohesion is part of the causal chain between environmental and built characteristics of a neighbourhood and health, with increasing mediation effect over time for physical health. An implication is that social mechanisms should be considered when studying the effect of neighbourhood characteristics on health inequalities. Moreover, our work positions social cohesion as a legitimate target of public health interventions at neighbourhood level.

## Data Availability

All information about the public availability of the SOEP data can be found at: https://www.diw.de/de/diw_01.c.615551.de/forschungsbasierte_infrastruktureinrichtung__sozio-oekonomisches_panel__soep.html

## Abbreviations

SOEP: German Socio-Economic Panel
MCS: Mental component score
PCS: Physical component score
